# Pediatric immunotherapy and HIV control

**DOI:** 10.1097/COH.0000000000000857

**Published:** 2024-05-02

**Authors:** Tehillah T. Chinunga, Ann Chahroudi, Susan P. Ribeiro

**Affiliations:** a Program in Immunology and Molecular Pathogenesis, Graduate Division of Biological and Biomedical Sciences, Laney Graduate School, Emory University; b Department of Pediatrics, Emory University School of Medicine; c Center for Childhood Infections and Vaccines of Children's Healthcare of Atlanta and Emory University; d Pathology Advanced Translational Research Unit (PATRU), Department of Pathology and Laboratory Medicine, Emory University School of Medicine; e Emory Vaccine Center; f Winship Cancer Institute of Emory University, Atlanta, Georgia, USA

**Keywords:** bNAbs, immune system, immunotherapy, pediatric HIV, virologic control

## Abstract

**Purpose of review:**

Highlighting opportunities/potential for immunotherapy by understanding dynamics of HIV control during pediatric HIV infection with and without antiretroviral therapy (ART), as modeled in Simian immunodeficiency virus (SIV) and Simian-human immunodeficiency virus (SHIV)-infected rhesus macaques and observed in clinical trials. This review outlines mode of transmission, pathogenesis of pediatric HIV, unique aspects of the infant immune system, infant macaque models and immunotherapies.

**Recent findings:**

During the earliest stages of perinatal HIV infection, the infant immune system is characterized by a unique environment defined by immune tolerance and lack of HIV-specific T cell responses which contribute to disease progression. Moreover, primary lymphoid organs such as the thymus appear to play a distinct role in HIV pathogenesis in children living with HIV (CLWH). Key components of the immune system determine the degree of viral control, targets for strategies to induce viral control, and the response to immunotherapy. The pursuit of highly potent broadly neutralizing antibodies (bNAbs) and T cell vaccines has revolutionized the approach to HIV cure. Administration of HIV-1-specific bNAbs, targeting the highly variable envelope improves humoral immunity, and T cell vaccines induce or improve T cell responses such as the cytotoxic effects of HIV-1-specific CD8^+^ T cells, both of which are promising options towards virologic control and ART-free remission as evidenced by completed and ongoing clinical trials.

**Summary:**

Understanding early events during HIV infection and disease progression in CLWH serves as a foundation for predicting or targeting later outcomes by harnessing the immune system's natural responses. The developing pediatric immune system offers multiple opportunities for specific long-term immunotherapies capable of improving quality of life during adolescence and adulthood.

## INTRODUCTION

**FIGURE 1:**
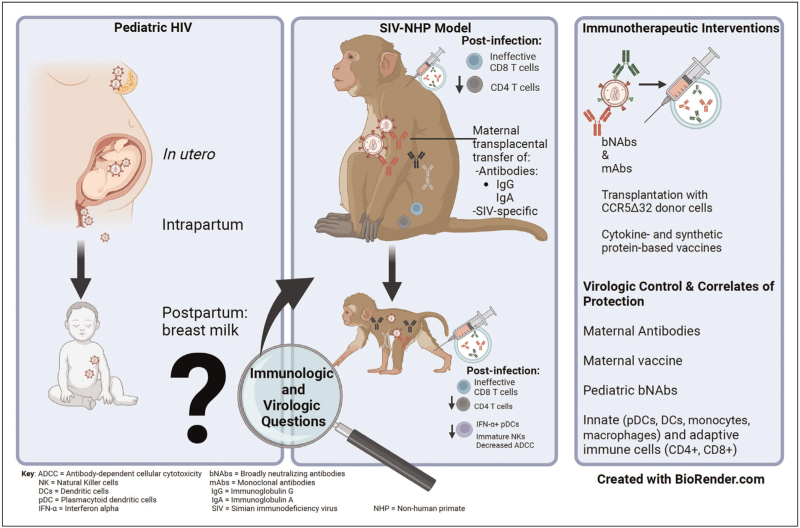
Overview. Mode of transmission, macaque model, pathogenesis of pediatric SIV, immunotherapeutic interventions, virologic control, and correlates of protection.

The global burden of HIV infection persists and in 2022, out of approximately 39 million people living with HIV (PLWH), 1.5 million were children living with HIV (CLWH) under the age of 15 [1]. An overview of the main areas of focus in this article is illustrated in figure 1. Vertical transmission of HIV is the most common route of infection for children, with high maternal viremia being the strongest predictor of transmission [1,2]. Additional factors such as settings in which there is a higher rate of HIV infections in women of child-bearing age, inadequate access to interventions (e.g., antiretroviral therapy [ART]), and unpredictable timing of ART initiation in pregnancy contribute to vertical transmission of HIV [2,3]. In the absence of ART during pregnancy, the rate of HIV transmission varies from 35% *in utero*, to 65% intrapartum and 7% to 22% through breastfeeding [4,5]. Without treatment, CLWH demonstrate a greater risk of acquiring opportunistic infections as well as more rapid disease progression compared to adults [3]. Investigating HIV infection in CLWH involves the understanding of the pediatric immune system and how it changes phenotypically and functionally in the neonatal, infant, and childhood periods. Within this framework, HIV pathogenesis, persistence, and host immune responses have been described, although much remains to be learned. Strategies to promote antiviral immune responses against HIV may be particularly beneficial for CLWH who tend to have less robust immunity. However, the type of immunotherapy that provides benefit may be highly age-dependent and HIV-related factors (time of ART initiation in relation to infection, duration of viral suppression, and so on) also influence outcomes. Well controlled studies of the immune system in CLWH, clinical trials of candidate immunotherapies, and descriptions of unique cases of protection inform our understanding of the desired immune features to induce better disease outcomes, reservoir reduction, as well as viral control without ART. Contributing to such scenarios, using pediatric nonhuman primate (NHP) models brings advantages to answer immunological questions as well as to assess the potential of immunotherapeutic interventions in impacting the size of the viral reservoir and/or control of viral rebound in cases of ATI (analytical treatment interruption). 

**Box 1:**
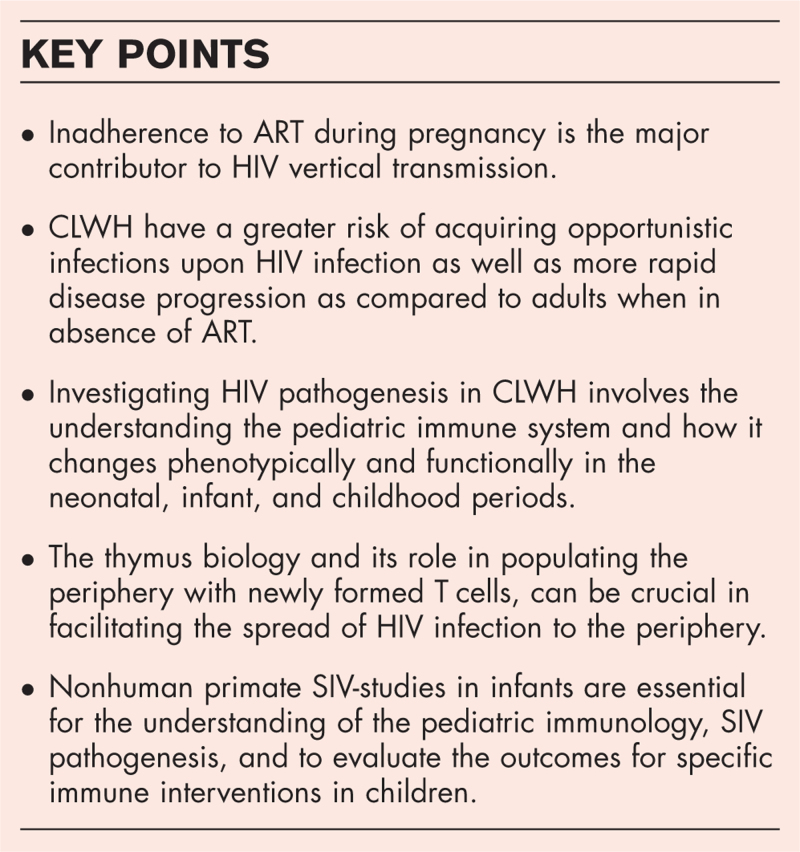
no caption available

## PATHOGENESIS OF PEDIATRIC HIV

The faster disease progression that follows vertical transmission, as opposed to horizontal transmission, is characterized by rapid depletion of CD4^+^ T cells, abnormal CD8^+^ T cell function, recurrent co-infections, and death within 2 years in half of untreated CLWH [3,6]. Innate immune cells such as mucosal macrophages, dendritic cells, and natural killer (NK) cells are also depleted during HIV infection in children. CLWH present a variety of clinical manifestations including persistent fever, chronic diarrhea, HIV encephalopathy and opportunistic infections such as Tuberculosis [7,8]. Much of the intensified pathogenesis of HIV in children stems from the fact that adaptive immunity is less effective in early life. Understanding the development and link between the innate and adaptive immune components is of interest when considering the pathogenesis of pediatric HIV [9]. Azzoni *et al.*[10] conducted a comprehensive study of 40 CLWH who acquired HIV-1 perinatally and were on ART, finding partial reconstitution of NK cells, plasmacytoid dendritic cells (pDCs) and myeloid DCs in virally suppressed CLWH in conjunction with CD4^+^ T cell reconstitution. The innate cell reconstitution in CLWH is distinct from what has been observed in adults [10,11]. However, despite the immune reconstitution, effector functions were still impaired as characterized by absence of mature NK cells, decreased IFN-α production by pDCs, as well as decreased IFN-γ production and antibody-dependent cytotoxic activity (ADCC) in both virally suppressed and viremic CLWH [10]. Further, HIV evades the immune system by impairing the function of CD4^+^ T cells as well as uninfected CD8^+^ T cells [3,6,12].

## INFANT IMMUNE SYSTEM

### Immune tolerance

Two unique features of the pediatric immune system, early in HIV infection before switching to increased immune activation, include a tolerogenic immune environment associated with low expression of the HIV co-receptor CCR5 and increased de-novo HIV variant-specific CD8^+^ T cell repertoires [13–16]. The unique attributes of the immune system of infants highlight that the immune development in early stages of life provides an opportunity to understand HIV infection in this window, when there is a difference in cell phenotype, target cells, immune tolerance, reduced immune activation and memory T cell pool, as well as distinctive responses to ART [17] as compared to adults. Due to the sterile in-utero environment in which an embryo develops, immune tolerance is key to preventing uncontrolled inflammation and maintaining the pregnancy [3,18]. Responses to antigenic stimuli after birth regulate the switch from tolerance towards more effector immune responses [18]. Competence of the infant immune system has been a topic of debate for many years, including questions regarding whether and how the immune systems of fetus and infants respond to pathogens or other stimuli. Mold *et al.*[19] demonstrated that induction of fetal regulatory T cells (Tregs), by transplacentally transferred maternal cells or antigens, creates a suppressive immune environment and antigen-specific tolerance during pregnancy. This tolerogenic fetal immune environment, while beneficial in preventing development of antimaternal antigen antibodies, can lead to increased susceptibility to infection with intracellular pathogens, including HIV [20]. Pregnancy is associated with an increase in the differentiation of CD4^+^ T cells into T helper 2 (Th2) cells over T helper 1 (Th1) cells and their respective cytokines [21–23]. Therefore, the Th1:Th2 balance is skewed towards Th2. Moreover, CD8 T cells are also skewed in their profile [21]. Contrary to the skew towards a tolerogenic environment with Tregs and Th2 *in utero* is a switch towards Th1 postbirth [24]. Addressing the role of the immune system in protecting against new infections and the spreading of the latent HIV reservoir is essential to the field.

### Thymus

The thymus is essential for T cell development and guiding mechanisms of detection of self- from nonself. An important step in T cell development is the formation of a T-cell receptor (TCR) through gene segment recombination events, followed by positive selection of T cells that weakly interact with self-peptides presented in major histocompatibility complexes (MHCs) on thymic antigen presenting cells and then negative selection which selects against strongly self-reactive T cells [25]. Thymocytes develop into naive T cells once they exit the thymus and await an encounter with antigen to differentiate into effector T cells. Naive T cells migrate to the periphery to populate lymphoid organs such as spleen, lymph nodes, and tonsils, as well as mucosal sites and the skin [25,26]. Given the role of the thymus in populating the periphery with newly formed T cells, its role in facilitating the spread of HIV infection has been an area of interest. HIV-exposed uninfected infants and infants with HIV infection experience a decrease in cortical double-positive (CD4^+^CD8^+^) T cells in the thymus as well as thymic output of naive CD4^+^ T cells [27,28].

## ANTIRETROVIRAL THERAPY ASSOCIATED VIROLOGIC CONTROL IN PEDIATRIC INFECTION

ART-free control of viremia is one of the ultimate goals towards HIV cure. Studies of the role of early ART administration in CLWH demonstrate improved virologic control and restriction of reservoir establishment, leading to a hypothesized contribution to ART-free viral control in CLWH, albeit at the expense of HIV-specific T cell responses [29–36]. Between 2005 and 2013, the Children with HIV Early Antiretroviral Therapy (CHER) phase III randomized trial was conducted in infants living with HIV from ages six to twelve weeks and assigned to ART when their CD4^+^ percentage decreased to less than 20 or 25% as well as according to established clinical criteria versus immediate ART [37]. The results demonstrated 75% reduction in mortality and HIV-1 disease progression with immediate ART [37]. In 2013, a case study of what is known today as the Mississippi baby, revealed that very early ART administration 30 h postbirth significantly disrupted establishment of HIV-1 reservoirs in the setting of a high-risk exposure and in-utero infection [30]. At 18 months of age, ART was discontinued, and surprisingly, HIV-1 antibodies, plasma HIV-1 RNA, and proviral DNA in peripheral blood mononuclear cells (PBMCs) remained undetectable until 30 months of age [30]. This finding demonstrates a protective role of very early ART in infants during a critical window which HIV reservoir is established. A subsequent study conducted in Botswana also revealed important features of viral reservoirs and immune responses in very early treated infants with in-utero HIV infection [38^▪▪^,^39^]. The IMPAACT P1115 trial is a phase 1/2 study assessing whether early ART in neonates restricts the HIV-1 reservoir with the end goal of ART-free remission. Administration of ART, after in-utero acquisition of HIV-1 is attainable and can reduce the size of the reservoir [40^▪▪^]. Latency and Early Neonatal Provision of Antiretroviral Drugs Clinical Trial [41] was a phase 4 study in South Africa, comprising 63 neonates diagnosed with HIV-1 less than 48 h of birth and promptly placed on ART [41,42]. Within 48 weeks of treatment, neonates who were treated very early with ART, demonstrated an inverse correlation between HIV-1 DNA and higher CD4^+^ T cells as well as viral load less than 100 000 copies/ml pre-ART [41,42]. The RV475/HIVNAT209 longitudinal clinical trial in Thailand, assessed whether early ART could serve as a prophylaxis and also reduce the HIV-1 reservoir size in 85 infants, followed for up to 3 years, who vertically acquired HIV-1 [43]. Prior to ART initiation, msRNA-producing cells and HIV DNA were significantly lower in infants who continued to receive ART postbirth compared to infants who never started or stopped [43]. The Children with HIV Early Antiretroviral Therapy (CHER) trial was phase 3 randomized clinical trial aimed at comparing the effects of anti-HIV drug regimens of different lengths in infants who acquired HIV at birth [37,44]. Moreover, the study sought to reduce infant mortality associated with HIV-1 in regions of high seroprevalence of HIV-1. Both early HIV diagnosis and ART significantly reduced early infant mortality by 76% and HIV progression by 75% [37]. Interestingly, the CHER trial identified a rare case of long-term virologic control in one South African child who at 9.5 years of age showed characteristics similar to uninfected children post-ART cessation [31,37]. HIV-1 standard diagnostic tests from the child were seronegative and he had a high CD4:CD8 ratio, as well as both low T cell activation and CCR5 expression [31]. Moreover, weak HIV-specific antibodies and CD4^+^ T cell responses were detected in the child [31]. In adolescents, an example of ART-free remission and virologic control of HIV-1 is the French teenager who acquired HIV-1 infection at birth, initiated ART 30 h after birth and lived in good health for 12 years post-ART cessation [45]. These examples demonstrate the undisputed benefits of early ART in reducing the size of, or restricting establishment of the long-persisting HIV-1 reservoir. There is a knowledge gap on both cellular and anatomic composition of the HIV reservoir as well as reasons for variability of virologic control in the pediatric population, hence the need for specific and reliable animal models to address underlying questions in the pediatric population living with HIV [17] is of great value.

## INFANT MACAQUE MODEL FOR IMMUNOTHERAPY

Nonhuman primates have long been used to study HIV because of the similarity of simian lentiviral pathogenesis and the immune response to that of HIV in humans, as well as giving an opportunity to focus on specific periods of infection and administer targeted therapeutic interventions [46]. Moreover, despite the disadvantages of cost and limited availability, NHP models allow for investigations of host genetics and control of environmental conditions to test hypotheses relevant to human biology and disease [47]. Simian immunodeficiency viruses (SIVs) and Simian human immunodeficiency viruses (SHIVs) are widely used for HIV/AIDS research [48,49]. While not the primary subject of this review, natural hosts for SIV (e.g., African green monkeys, Sooty Mangabeys, mandrills and chimpanzees) have informed our understanding of HIV pathogenesis and vertical transmission [49–52]. To mimic HIV-1 infection, nonnatural hosts such as Asian rhesus macaques are infected with SIV strains that induce high viral loads, progressive CD4^+^ T cell depletion and opportunistic infections [49,53]. Challenges to using the SIV/macaque model also exist, including lack of sensitivity to drugs that inhibit key HIV enzymes such as reverse transcriptase, inability to test HIV Env-specific immunogens, and difference in co-receptor usage [49,54].

SHIV chimeras incorporating an HIV Env into an SIV backbone have been useful in measuring the efficacy of approaches focused on passive administration or induction of anti-HIV antibodies. In one example, Jayaraman *et al*. [55] developed a pig-tailed macaque model of vertical transmission in which ten pregnant dams were intravenously infected with SHIV-SF162P3 in their second trimester. Passive transfer of anti-HIV antibodies was reported in plasma of infants at birth until at least 5 weeks, and maternal neutralizing antibodies were proposed to have influenced the rate of vertical transmission as well as pathogenesis following infection [56^▪^]. These results led to the hypothesis that purposely inducing more and specific maternal antibodies through vaccination during pregnancy could provide protection to exposed infants. Van Rompey *et al.*[57] conducted a study to elucidate the role of transplacental transfer of SIV-specific maternal antibodies from vaccinated pregnant dams to infants exposed to SIV postnatally. Antiviral IgG or IgA antibodies elicited by the prime-boost vaccination regimen (prime: live-attenuated SIVmac1A11 / boost: inactivated SIVmac1A11) were associated with protection against mucosal SIV infection in the infants [57]. Other maternal vaccination approaches have not shown a protective effect on infant oral SHIV acquisition despite induction of robust Env-specific IgG and durable milk IgA responses [58].

Immunization of newborn macaques has also been tested as a means to prevent breast milk transmission [59]. While unsuccessful in terms of protection, infants vaccinated with oral vesicular stomatitis virus (VSV) expressing SIV genes followed by modified vaccinia virus Ankara (MVA) expressing SIV genes developed SIV-specific plasma IgA during challenge and levels were inversely correlated with peak viremia [60]. Additionally, infant macaques with low viremia had a greater abundance of SIV-specific TNF-α or IFN-γ-producing CD8^+^ T cells and central memory CD4^+^ T cells, with significant depletion of Tregs in tonsils [60]. Similarly, orally administered Mycobacterium tuberculosis expressing SIV Env/Gag followed by MVA-SIV Env/Gag/Pol did not protect from SIV acquisition in infant macaques, although a subset of controllers showed maintenance of CD4^+^ T cells and an inverse correlation between viremia and SIV Env-specific salivary and intestinal immunoglobulin A (IgA) along with SIV Env-specific plasma IgG of high avidity prechallenge [61]. These studies indicate that antiviral antibodies and effector CD8^+^ T cells along with absence of Tregs may contribute to vaccine-elicited viral control (Fig. 1).

Using vaccination in the setting of established infection and ART has also been studied as an immunotherapeutic approach to control viral rebound following ART interruption [62]. To consider additional immunologic factors associated with viral suppression in CLWH, Evangelous and colleagues conducted a study in ART-naïve SHIV.CH848.10.17 DT.E169K-infected infant rhesus macaques that spontaneously controlled viremia. They identified reduced levels of several pro-inflammatory cytokines in addition to a profile of activated and/or cytotoxic CD8^+^ T cells, NK cells, and monocytes in the controllers [56^▪^].

Passive administration of HIV-specific antibodies, including broadly neutralizing antibodies (bNAbs), was also initially studied in NHP models. To develop prophylaxis strategies against perinatal HIV infection, four doses of the HIV bNAbs PGT121 and VRC07–523 were given to infant rhesus macaques starting 1 day after oral exposure to SHIV-SF162P3. This passive immunotherapy prevented replication of SIV in blood and tissues for 6 months after exposure [63]. A follow-up study from the same group demonstrated that a single dose of the same HIV bNAbs given within 30 h after oral inoculation with SHIVSF162P3 rendered 100% of infant macaques aviremic in the absence of adaptive immunity [39]. These results led to several clinical trials evaluating HIV bNAbs in children at a high risk for HIV acquisition (see next section). Polyclonal antibodies have also been studied in NHPs due to the theoretical advantage of targeting a diverse range of HIV-1 epitopes [62,64–66].

## STATE OF CURRENT IMMUNOTHERAPY APPROACHES IN CHILDREN

Immunotherapy in the field of HIV aims to build HIV-specific immunity, rescue immune exhaustion, target latent reservoir eradication, and prevent or control viral rebound [67] upon ART cessation. In addition to early ART, immune-based HIV cure strategies also include T-cell, antibody-based, cytokine, and immune checkpoint blockade therapies in people with HIV (mostly adults) [68–71]. In CLWH and HIV-exposed uninfected (HEU) children, the main immunotherapeutic approaches that have been pursued include vaccines and bNAbs. In ART-treated CLWH compared to ALWH, a clinical trial was conducted to investigate the ability of an HIV DNA vaccine to induce HIV-specific cellular immunity as well as reduce HIV reservoirs [72]. Ten children between 4 and 16 years of age on ART were vaccinated while another group of ten served as controls with both groups maintaining ART during and after immunizations. In parallel, 10 ALWH on ART between 29 and 56 years of age were vaccinated., with appropriate controls assigned [72]. In both CLWH and ALWH, the HIV DNA vaccine induced new HIV-specific cellular immune responses specific to HIV Gag antigens; however, no benefit for reservoir decay was observed [72]. The Safety and Effects of Using Prime-boost HIVIS DNA and MVA-CMDR Vaccine Regimens with or without Toll-like Receptor 4 Agonist on HIV Reservoirs in Perinatally HIV Infected Children and Youth (HVRRICANE) study is currently recruiting [73]. HVRRICANE is a randomized phase 4 clinical trial recruiting participants that are at least 9 years old who acquired HIV-1 perinatally and initiated ART before 6 months of age [73]. The goal is to evaluate the safety and effects of prime-boost HIVIS DNA and MVA-CMDR vaccine treatments in the presence or absence of TLR4 agonist and how this impacts HIV reservoir [73]. BNAbs are also an attractive immunotherapeutic option in pediatric HIV. During HIV-1 infection, approximately 1% of PLWH develop broad and potent neutralizing antibodies in contrast to the majority of PLWH who develop virus strain-specific autologous neutralizing antibodies [63,74–76]. Interestingly, a high frequency of V2-apex bNAbs was observed in infants with cross-clade neutralization in a cross-sectional study of 51 ART-naive infants with HIV-1 clade C [63], highlighting a unique aspect of the immune response to HIV in children. The Tatelo study was a clinical trial established to evaluate the role of dual bNAbs (VRC01LS and 10–1074) as an alternative treatment, in children who initiated ART treatment before 7 days of age until at least 96 weeks [63]. Almost half of the children who received ART from birth maintained viral suppression for 24 weeks with monthly administration of the two bNAbs. In addition to the success of the bNAbs in viral suppression, the authors identified predictive markers of treatment success. Markers identified as predictors of success include levels of HIV DNA in peripheral blood mononuclear cells (PBMCs), viral suppression before treatment, and HIV qualitative DNA test at the entry of the study vs. no positive serology result by enzyme immunoassay (EIA) [63]. Table 1 summarizes a summary of additional clinical trials focusing on immunotherapy in CLWH [77–85].

**Table 1:** Summary of completed and ongoing clinical trials in children living with HIV (CLWH) grouped by immunotherapeutic approach

Category	Study	Drug/Intervention	Age	Status	Goal	Main result
Pathogenesis	Evaluation of the HIV-1 Reservoir in the Central Nervous System of Perinatally-Infected Youth and Young Adults With Cognitive Impairment (NCT03416790)	None	13–24 years	Completed	To measure the central nervous system (CNS) reservoir by determining the frequency of HIV in cerebral spinal fluid (CSF) and correlation with inflammatory as well as neuronal injury markers.	To be determined
Antibodies	Evaluating the Safety and Antiviral Activity of Monoclonal Antibody VRC01 in Infants With HIV Receiving Combination Antiretroviral Therapy (NCT03208231)	Human IgG1 mAb (VRC01)	0–12 weeks	Completed	To evaluate the safety and antiviral activity of VRC01 as well as in promoting clearance of HIV-1 infected cells in infants living with HIV beginning combination antiretroviral therapy.	To be determined
	Analytical Treatment Interruption (ATI) to Assess the Immune System's Ability to Control HIV in Participants Who Became HIV-infected During the HVTN 703/HPTN 081 AMP Study (NCT04860323 and NCT04801758)	Human IgG1 mAb (VRC01) [76–78]	Child, adult, older adult (could not find the exact age range)	Recruiting and active	To evaluate viral and immune system responses during ATI in participants living with HIV, who received VRC01 or placebo	To be determined
	Very Early Intensive Treatment of HIV-Infected Infants to Achieve HIV Remission (NCT02140255)	Four antiretroviral drugs (Nucleoside Reverse Transcriptase Inhibitors (NRTIs), Nevirapine (NVP), Lopinavir/Ritonavir (LPV/r), Raltegravir [39] and a monoclonal antibody (VRC01)	Up to 10 Days	Recruiting	To determine the effects of early intensive ART on achieving HIV remission in infants living with HIV.	To be determined
	Evaluating the Safety and Pharmacokinetics of VRC01, VRC01LS, and VRC07–523LS, Potent Anti-HIV Neutralizing Monoclonal Antibodies, in HIV-1-Exposed Infants (NCT02256631)	Anti-HIV neutralizing mAbs (VRC01, VRC01LS, and VRC07–523LS)	0 days to 5 days	Completed	To assess the safety and pharmacokinetics (PK) of three mAbs, VRC01, VRC01LS, and VRC07–523LS, in infants exposed to HIV who are at increased risk of vertical acquisition of HIV.	Administration of VRC01 as single or multiple doses was well tolerated in the infants [79]. Further studies are required to determine prevention of HIV transmission.
	Dual bNAb Treatment in Children (NCT03707977)	Dual bNAb combination (VRCO1LS and 1074)	96 weeks to 7 years	Completed	To evaluate the role of dual bNAbs (VRC01LS and 10–1074) as an alternative treatment for the maintenance of HIV suppression in CLWH who initiated ART early.	Almost half of the children who received ART from birth maintained viral suppression for 24 weeks with monthly administration of the two bNAbs. The levels of HIV DNA in peripheral blood mononuclear cells (PBMCs), viral suppression before treatment, and HIV qualitative DNA test at the entry of the study were identified as predictors of success [37].
Vaccine	Evaluating Safety and Immune Response to the HIV-1 CH505 Transmitted/Founder gp120 Adjuvanted With GLA-SE in Healthy, HIV-exposed Uninfected Infants (HVTN 135) (NCT04607408)	A protein vaccine (CH505TF gp120) which has been tested in adults [85] and a synthetic TLR4 agonist (Glucopyranosyl Lipid) as an adjuvant. [80]	0 days to 5 days (Child)	Phase I, Active, not recruiting	To evaluate the safety and immune response in healthy HIV-exposed and uninfected infants to the protein vaccine, CH505TF gp120, alongside the GLA-SE adjuvant.	To be determined. Maternal antibodies will also be quantified from mother-infant pairs.
	A Phase I Study to Evaluate the Safety and Immunogenicity of Recombinant HIV-1 Envelope Antigen in Children Born to HIV-Infected Mothers (NCT00000774)	Envelope recombinant proteins vaccines (rgp120/HIV-1MN (Genentech) and rgp120/HIV-1SF2 (Chiron/Biocine)	1 day to 3 days	Completed	To evaluate the safety of envelope recombinant proteins rgp120/HIV-1MN and rgp120/HIV-1SF2 in infants of unknown HIV status born to MLWH. Subsequently, changes in viral load in infected infants and absolute CD4^+^ T cell counts in all immunized infants [81,82].	Responses to heterologous HIV-1 envelope antigens were detected [81]. Moreover, the vaccines were well tolerated in the study population, with no evidence of accelerated immunologic decline [82].
	Safety and Effectiveness of CD4-IgG2 in HIV-Positive Children (NCT00000876)	Synthetic protein-based vaccine (CD4-IgG2)	2–12 years	Completed	To determine whether administering a synthetic protein called CD4-IgG2, is safe and effective in CLWH. CD4-IgG2 blocks the entrance of HIV into CD4^+^ T cells [84].	The treatment was well tolerated and capable of reducing HIV-1 burden as observed by 28 days posttherapy, the peak HIV-1 cellular infectious units was reduced in all six children [78].
	Evaluating Safety and Immune Response to the HIV-1 CH505 Transmitted/Founder gp120 Adjuvanted With GLA-SE in Healthy, HIV-exposed Uninfected Infants (HVTN 135) (NCT04607408)	A protein vaccine (CH505TF gp120) which has been tested in adults [86] and a synthetic TLR4 agonist (Glucopyranosyl Lipid) as an adjuvant [80]	0–5 days (Child)	Phase I, Active, not recruiting	To evaluate the safety and immune response in healthy HIV-exposed and uninfected infants to the protein vaccine, CH505TF gp120, alongside the GLA-SE adjuvant.	Maternal antibodies will be quantified from mother-infant pairs
	A Placebo-Controlled, Phase I Clinical Trial to Evaluate the Safety and Immunogenicity of Recombinant Envelope Proteins of HIV-1 gp160 and gp120 in Children >= 1 Month Old With Asymptomatic HIV Infection (NCT00000762)	gp160 (MicroGeneSys), rgp120/HIV-1MN (Genentech), and rgp120/HIV-1SF2 (BIOCINE) and adjuvants	1 month to 18 years (Child, Adult)	Completed	To evaluate the safety and immunogenicity of gp160, rgp120/HIV-1MN, and rgp120/HIV-1SF2 as well as their adjuvants in CLWH between 1 month and 18 years of age [83].	No adverse events. Between 30 and 56% of volunteers exhibited a lymphoproliferative response to vaccine antigens; 65% of vaccinees but none of placebo recipients exhibited moderate or strong responses to HIV specific antigens. The rgp160 and gp120 subunit vaccines were safe and immunogenic in this population. [83].
	A Study of the Effects of Giving Two Anti-HIV Vaccines to Babies of HIV-Positive Mothers (NCT00000879)	Anti-HIV vaccines (LVAC vCP1452 alone or with AIDSVAX B/B)	0–3 days (Child)	Completed	To determine whether administering ALVAC vCP1452 anti-HIV vaccine alone or with another vaccine called AIDSVAX B/B to babies of MLWH is safe and how the vaccines affect the baby's immune system to reduce the risk of HIV acquisition.	Maternally acquired antibodies were measured but they waned quicker than vaccine induced Env-specific IgG responses.
Other Immunotherapies	Interleukin-2 Plus Anti-HIV Therapy in HIV-Infected Children With Weakened Immune Systems (NCT00006066)	IL-2 and Plus Anti-HIV Therapy in HIV-Infected Children With Weakened Immune Systems	2 years to 18 years (Child, Adult)	Completed	To determine the safety and best dose of a interleukin-2 (IL-2) drug given with anti-HIV therapy in CLWH.	To be determined
	Safety, Tolerability, and Anti-HIV Activity of PEG-Intron in HIV-Positive Children (NCT00006325)	Interferon alpha-based anti-HIV drug (Peginterferon alfa-2b)	3 months to 16 years	Completed	To determine whether PEG-Intron is tolerated when given to CLWH. In addition to investigating how much gets into and stays in the blood while observing how well PEG-Intron works to reduce viral load.	The vaccine was safe, however, there was no effect on the clinical course of the participants over 48 months of study. Moreover, due to small sample size efficacy of HIV vaccine as an immunotherapy option was not feasible.

## CONCLUSION

NHP studies and clinical trials have facilitated probing complex immunologic questions targeted toward the generation of HIV-specific humoral and cellular responses capable of controlling viremia and eliminating infected cells. Even though early ART in CLWH promotes virologic control as well as reduces reservoir size, challenges ensue due to the compromised antiviral immune response. Moreover, many immunotherapeutic strategies tested in ALWH are yet to be conducted in CLWH. The current body of evidence has yet to fill the knowledge gap of how to reproducibly induce ART-free viral control in the context of a developing immune system.

## Acknowledgements

*Department of Pathology and Department of Pediatrics at Emory University for the support.*

### Financial support and sponsorship

*P01 HD112217, P30AI050409, UM1AI16456.*

### Conflicts of interest

*There are no conflicts of interest.*

## REFERENCES AND RECOMMENDED READING

Papers of particular interest, published within the annual period of review, have been highlighted as:

▪ of special interest
▪▪ of outstanding interest
